# The Medical Society of the State of New York

**Published:** 1892-06

**Authors:** 


THE MEDICAL SOCIETY OF THE STATE OF NEW YORK.—*Official Bulletin.*—The business committee of this society has been organized for the meeting of February, 1893, by the appointment of Dr. Seneca D. Powell, of No. 12 West Fortieth street, New York City, Chairman, and Drs. William Maddren, of Brooklyn, and John O. Roe, of Rochester, associates. The programme for the scientific work of the session is already well advanced in its preparation.

